# Ataxia-Telangiectasia patients get a rare chance to meet the experts at a dedicated workshop in IFOM (the FIRC Institute of Molecular Oncology)

**DOI:** 10.3332/ecancer.2017.ed66

**Published:** 2017-05-08

**Authors:** Linda Cairns

**Affiliations:** Science Editor, *e*cancermedicalscience, European Institute of Oncology, Milan, Italy

**Keywords:** Ataxia telangiectasia, immunodeficiency, cancer predisposition, cerebellar degeneration

## Abstract

Ataxia telangiectasia (A-T) is a genetic syndrome characterized by cerebellar degeneration, telangiectasia, immunodeficiency and cancer predisposition. A-T occurs in between 1 in 40,000 and 1 in 100,000 live births. The first symptoms normally occur in early childhood when the infant begins to walk. Affected children have immunodeficiency and an increased predisposition for cancers. A-T is caused by mutations in the *ATM* (Ataxia Telangiectasia, Mutated) gene which encodes a protein of the same name.

Ataxia-telangiectasia (A-T) is a rare inherited disorder that affects the nervous system, the immune system and cancer predisposition. A-T is a life-shortening disease which is usually diagnosed in early childhood. At present there is no cure. A major problem with rare diseases is the lack of funding for research compared to more common diseases.

A-T is caused by defects in the ATM gene. Roughly 1 in 250 individuals carries a defective copy of this gene (healthy carriers). The problem arises if two carriers have a child; there is a 25% chance that the child will have A-T. The ATM gene controls how the body produces a protein that helps control cell division and is important for the normal physiology of the nervous system and immune system. The ATM protein is also able to recognize damaged DNA and to coordinate DNA repair. Failure to efficiently repair damaged DNA is a hallmark of cancer.

Mutations in the ATM gene decrease or eradicate the function of the ATM protein and the cells become unstable and die. The cells of the cerebellum which co-ordinate movement are particularly affected by loss of the ATM protein. The loss of these brain cells causes the movement problems characteristic of ataxia-telangiectasia. The inability of ATM defective cells to respond correctly to DNA damage allows breaks in DNA strands to accumulate and can lead to the formation of cancerous tumors.

Although research into this rare condition has made significant advances in recent years there is still a lot to learn. We don’t know why it affects some bodily processes but not others or why some cells in the body die without the ATM protein yet others survive. There is valuable research currently under way and the International Ataxia-Telangiectasia Workshop held at IFOM (the FIRC Institute of Molecular Oncology) in Milan this year brought together the most renowned researchers in the field. This unique conference focused on the various aspects of ATM biology from the basic mechanisms underlying the function of ATM and its related proteins to the more clinical aspects of Ataxia-Telangiectasia. Topics included: A-T clinical aspects and therapeutic options; ATM, cellular metabolism and cancer; maintenance of genome stability.

An interactive “ask the expert” session provided a unique opportunity for A-T patients, families and advocates to have access to the latest scientific breakthroughs and also to ask questions and discuss disease related problems. The session, coordinated by William Davies, the Chief Executive of the A-T Society, was composed of a panel of international experts including Yossi Shiloh (Tel Aviv) who discovered ATM some 22 years ago and whose lab investigates the various branches of the ATM-mediated DNA damage response and Howard M Lederman (Johns Hopkins, USA) who discussed the importance of early diagnosis for genetic counseling, appropriate care, and avoidance of unnecessary tests.

The importance of this conference over the years is that discoveries made by scientists studying A-T have led the way towards our understanding of cancer. At this conference new results were presented linking malfunctions in DNA repair and the DNA damage response. A clear link emerged between DNA damage, neurodegeneration and aging as reported by many investigators, including Jan Hoeijmakers, who has pioneered DNA repair and aging studies. These new discoveries will potentially change the way we think about some brain diseases.

“One very positive observation about #atw2017 is that many presentations have a more holistic approach, linking the various functions of ATM- DNA repair, anti-oxidation, mitochondrial function, autophagy and so on. Much more so than a few years ago, when the focus seemed more on individual processes” said William Davies. “This is much more in line with the reality for people with A-T who experience everything together. It also gives me hope that we can make more rapid progress in understanding what causes A-T to develop as it does”.

